# Early and midterm results of valve-sparing aortic root reconstruction with a bovine pericardium patch for patients with acute type a aortic dissection

**DOI:** 10.3389/fcvm.2022.1009171

**Published:** 2022-11-02

**Authors:** Jue Yang, Xin Li, Meifen Wu, Jinlin Wu, Zerui Chen, Tucheng Sun, Ruixin Fan, Changjiang Yu

**Affiliations:** Department of Cardiac Surgery, Guangdong Cardiovascular Institute, Guangdong Provincial Key Laboratory of South China Structural Heart Disease, Guangdong Provincial People's Hospital, Guangdong Academy of Medical Sciences, Guangzhou, China

**Keywords:** valve-sparing, aortic root reconstruction, bovine pericardium patch, aortic dissection, early and midterm results

## Abstract

**Objectives:**

We evaluated the feasibility, effectiveness, and safety of valve-sparing aortic root reconstruction with a bovine pericardium patch for patients with acute type A aortic dissection (ATAAD).

**Methods:**

From January 2016 to January 2021, 895 consecutive patients with ATAAD received surgical treatment at our hospital. After applying our exclusion criteria, 508 patients were included in this research. Based on the attending surgeon's judgment and preference, 192 patients underwent our novel surgical novel surgical technique of valve-sparing aortic root reconstruction (repair group [RG]) and 316 patients underwent the Bentall procedure (Bentall group [BG]).

**Results:**

In the RG, the early mortality rate was 4.2% (8/192). In the BG, the early mortality rate was 5.1% (16/316). There were no significant differences between groups. The incidence of postoperative renal failure in the BG was significantly higher than that in the RG. The mean follow-up time was 2.93 years (standard deviation, ±1.38 years). There were no significant differences between groups regarding ATAAD-related mortality and reoperation. In the RG, the rate of freedom from aortic root reoperation at 5 years was 98.2%, and the grade of aortic regurgitation 6 months later was significant less than that before surgery, and it did not worsen during later follow-up.

**Conclusions:**

Valve-sparing aortic root reconstruction with a bovine pericardium patch can be successfully performed for selected patients with ATAAD and is associated with low in-hospital and late mortality rates and low root reoperation rates during early and midterm follow-up.

## Introduction

Acute type A aortic dissection (ATAAD) is a life-threatening disease that is associated with a very high risk of perioperative mortality (1, 2). This disease usually involves the aortic root and leads to aortic sinus dissection, aortic valve regurgitation, and coronary artery stenosis or occlusion (3–5). Several surgical strategies have been created to manage aortic root lesions with ATAAD, including aortic root replacement and aortic valve preservation with aortic root reconstruction (6–10).

For aortic root dissection, aortic root replacement, such as the Bentall procedure, has been the standard procedure and has improved the long-term prognosis for many patients, especially those with Marfan syndrome or aortic root aneurysm (6, 11). However, the lifelong anticoagulation therapy might be related to a poor quality of life and the risk of bleeding and thromboembolic complications. For most patients, the aortic annulus and aortic leaflets are not involved; therefore, reconstructive surgical techniques that preserve the valve leaflets in the aortic root are encouraged. Many aortic root reconstructive techniques have been created, such as traditional methods that include the use of gelatin resorcin formalin glue, fibrinous glue, and Teflon felt strips to repair the aortic sinus, aortic root remodeling technique (Yacoub procedure), and valve-sparing aortic root reimplantation (David procedure). However, the outcomes of these surgical techniques have not been satisfactory, and some of them (Yacoub procedure and David procedure) have not been popularized because of their significant complexity and steep learning curve (7–9, 12, 13). We introduce our novel technique of valve-sparing aortic root reconstruction with a bovine pericardium patch for patients with ATAAD and evaluate the feasibility, effectiveness, and safety of this technique.

## Patients and methods

### Study patients

From January 2016 to January 2021, 895 consecutive patients with ATAAD received surgical treatment at our hospital. Of those 895 patients, 793 patients had aortic sinus dissection. Of those 793 patients, 265 patients with dilation of the aortic sinus (maximum diameter, >45 mm), severe aortic regurgitation, severe coronary lesion caused by proximal dissection (Neri type C), organic disease in aortic valve leaflets, or connective tissue disorders and those who were indicated for aortic root replacement were excluded from the study population. Of the remaining 528 patients, twenty underwent traditional aortic root reconstruction (fibrinous glue and Teflon felt strips were used to repair the aortic sinus) or the David procedure; these patients were excluded from subsequent analyses because of the small population. Of the remaining 508 patients, 316 underwent the Bentall procedure and 192 underwent aortic valve resuspension and reinforcement of the aortic sinus with a bovine pericardium patch. These 508 patients were included in our research. The study was approved by the research ethics committee of Guangdong Provincial People's Hospital, and informed consent was obtained from all participants.

In our emergency department, all patients with ATAAD underwent contrast multidetector computed tomography (MDCT) and transthoracic echocardiography. The MDCT figures were used to measure the maximum diameter of the aortic sinus and classify the coronary lesion type (Neri type A, B, or C). Transthoracic echocardiography was used to classify aortic regurgitation (none, mild, moderate, or severe) and confirm whether there were organic diseases in the aortic valve leaflets.

**Figure 1:**
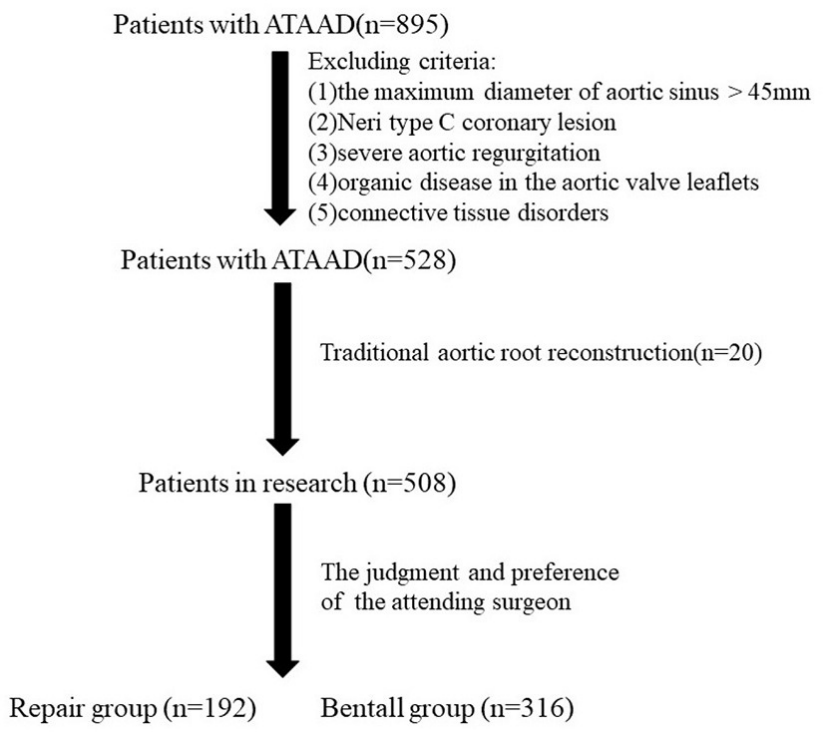
The study flow chart. ATAAD, acute type A aortic dissection.

Indications for our novel surgical technique of valve-sparing aortic root reconstruction with a bovine pericardium patch were as follows: aortic sinus with a maximum diameter <45 mm; Neri type A or B coronary lesion; none, mild, or moderate aortic regurgitation; no organic disease in the aortic valve leaflets; no connective tissue disorders; and the judgment and preference of the attending surgeon. Excluding criteria included the maximum diameter of aortic sinus > 45 mm; Neri type C coronary lesion; severe aortic regurgitation; organic disease in the aortic valve leaflets; and connective tissue disorders. The 192 patients who underwent our novel surgical technique of valve-sparing aortic root reconstruction comprised the repair group (RG). The 316 patients with aortic sinus with a maximum diameter <45 mm, Neri type A or B coronary lesion, none, mild, or moderate aortic regurgitation, no organic disease in the aortic valve leaflets, and no connective tissue disorders underwent the Bentall procedure based on the attending surgeon's judgment and preference comprised the Bentall group (BG). The study flow chart was demonstrated in Figure 1.

### Surgical procedure

We have introduced the details of establishment of cardiopulmonary bypass and cerebral perfusion in our previous research (14). All patients underwent replacement of ascending aortic and total aortic arch with an intraoperative stent inserted in the descending aorta. In some patients with valvular heart disease or coronary heart disease, cardiac valvular surgery and coronary artery bypass grafting were performed concomitantly.

**Figure 2:**
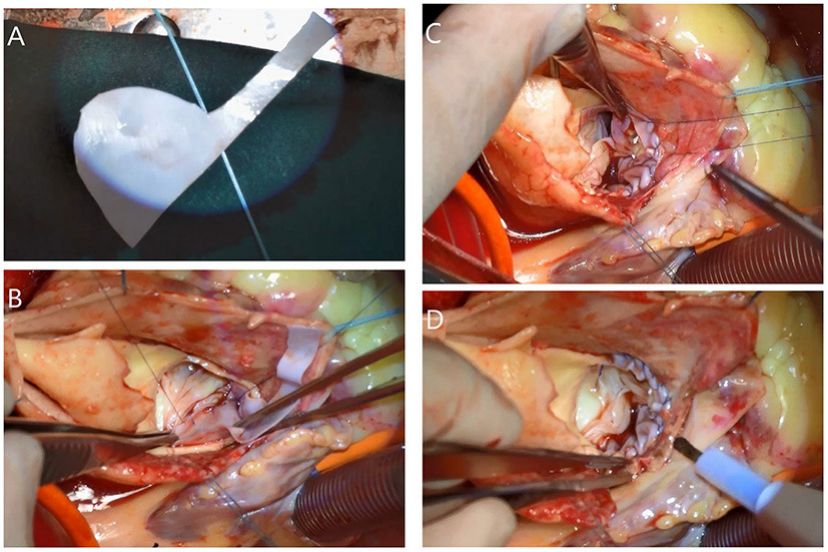
After resuspension of the affected commissure (right and noncoronary) of the aortic valve. **(A)** The bovine pericardium patch was created for the non-coronary sinus and sinotubular junction of the right sinus was placed internally. **(B)** The 5-0 polypropylene continuous sutures were applied around the edge of the non-coronary sinus and **(C)** around the sinotubular junction of the right sinus. **(D)** The reconstructed aortic sinus.

Aortic root reconstruction included resuspension of the aortic valve which was applied by using one to three pledged 5-0 polypropylene mattress sutures at the roof of the affected commissures of the aortic valve and performing our novel repair technique with a bovine pericardium patch. The patch comprised a strip of bovine pericardium (Balance Medical, Surgical Biopatch, Beijing, China) that was placed around the internal side of the whole non-coronary sinus to seal the dissection of the aortic sinus and strengthen the whole non-coronary sinus and sinotubular junction of the affected aortic sinus. This step must be completed carefully to avoid injuring or blocking the origin of the left and right coronary arteries. Then, 5-0 polypropylene continuous sutures were applied to fix the strip of bovine pericardium (the patch) and aortic wall around the edge of the non-coronary sinus and sinotubular junction of the affected sinus (Figure 2). If the coronary artery was affected by ATAAD (Neri type A or B), then we tried to repair it by using 6-0 polypropylene continuous sutures around the lesion area of the coronary ostia. If the repair failed, then coronary artery bypass grafting with a saphenous vein graft was performed. Finally, a proximal anastomosis with a woven dacron vascular graft was sewn to the reconstructed aortic root.

### Follow-up

We evaluated the postoperative mortality and complications during hospitalization of 508 patients. The early results were defined as the hospitalization results or those 3 months after surgery. After discharge, all patients participated in strict follow-up evaluations at 1, 3, 6, 12 months, and then yearly thereafter. The primary outcome was death and the secondary outcomes included bleeding, heart failure, cerebrovascular event, gastrointestinal hemorrhage, pneumonia, postoperative renal failure, multiple organ dysfunction syndrome, aortic root reoperation and severe aortic regurgitation. Follow-up in this study was conducted through outpatient examination and telephone interview and there was no missing case.

### Statistical analysis

Statistical analysis was performed using SPSS version 26.0 for Windows (SPSS, Chicago, IL). Categorical variables were expressed as frequencies and percentages. The χ^2^ test was performed for the univariate analysis in categorical variables. If *P* < 0.05, then we considered the difference to be significant. Continuous variables were expressed as medians or means ± standard deviations (SD). For the univariate analysis in continuous variables, firstly, the Kolmogorov–Smirnov test would be performed to determine whether these continuous variables were normally distributed. Then the Levene's test was performed. If *P* < 0.05, the Wilcoxon test would be performed for the univariate analysis. If *P* ≥ 0.05, the *t*-test would be performed for the univariate analysis. Finally, if P (the *t*-test or Wilcoxon test) <0.05, we considered the difference between two groups to be significant.

## Results

### Baseline characteristics

There were 122 males and 70 females in the RG, and their mean age was 52.4 years (SD, ±12.8 years; range, 25–75 years). Furthermore, 171 patients had a history of hypertension, seven patients were diagnosed with coronary artery disease, five patients had diabetes mellitus, two patients had chronic kidney disease, three patients had a history of stroke, and 10 patients underwent previous cardiac surgery (mitral valve repair, five patients; atrial septum defect repair, three patients; thoracic endovascular aneurysm repair, two patients). Coronary malperfusion, acute renal failure, bowel ischemia, lower limb ischemia, and cardiac tamponade were observed in 14 (7.3%), 3 (1.6%), 2 (1.0%), 7 (3.6%), and 13 (6.8%) patients, respectively. Using MDCT, we found that the maximum aortic diameter in the aortic sinus was 38.5 mm (SD, ±3.5 mm; range, 30.0–45.0 mm), and 73 patients had ATAAD of the coronary arteries (Neri type A, 55 cases; Neri type B, 18 cases). Using transthoracic echocardiography, we found that the left ventricular ejection fraction was 64.0% (SD, ±3.1%; range, 48.0–70.0%), and that the aortic regurgitation grades were as follows: none, 75 patients; mild, 80 patients; and moderate, 37 patients. All patients in this group underwent valve-sparing aortic root reconstruction with a bovine pericardium patch.

There were 207 males and 109 females in the BG, and their mean age was 53.9 years (SD, ±13.1 years; range, 25–84 years). Furthermore, 279 patients had a history of hypertension, 12 patients were diagnosed with coronary artery disease, 12 patients had diabetes mellitus, four patients had chronic kidney disease, four patients had a history of stroke, and 12 patients had undergone previous cardiac surgery (mitral valve repair, six patients; atrial septum defect repair, two patients; ventricular septum defect repair, one patient; thoracic endovascular aneurysm repair, three patients). Coronary malperfusion, acute renal failure, bowel ischemia, lower limb ischemia, and cardiac tamponade were observed in 25 (7.9%), 4 (1.3%), 4 (1.3%), 14 (4.4%), and 18 (5.7%) patients, respectively. Using MDCT, we found that the maximum aortic diameter of the aortic sinus was 39.0 mm (SD, ±2.9 mm; range, 32.0–45.0 mm), and that 109 patients had ATAAD of the coronary arteries (Neri type A, 80 cases; Neri B type, 29 cases). Using transthoracic echocardiography, we found that the left ventricular ejection fraction was 63.5% (SD, ±4.2%; range, 45.0–74.3%), and that the aortic regurgitation grades were as follows: none, 107 patients; mild, 134 patients; and moderate, 75 patients. All patients in this group underwent the Bentall procedure. There were no significant differences between these two groups in terms of preoperative clinical characteristics (Table 1).

**Table 1:** Preoperative clinical characteristics of the patients.

	**RG** **(*n* = 192)**	**BG** **(*n* = 316)**	**Kolmogorov–Smirnov test (RG/BG)**	**Levene's test**	***P*-value**
Age (y)	52.4 ± 12.8	53.9 ± 13.1	0.200/0.200	0.520	0.190
Sex (male/female)	122/70	207/109			0.653
Hypertension^a^	171 (89.1%)	279 (88.3%)			0.791
Grade 1	15 (7.8%)	22 (7.0%)			0.721
Grade 2	105 (54.7%)	160 (50.6%)			0.375
Grade 3	51 (26.6%)	97 (30.7)			0.320
Diabetes mellitus	5 (2.6%)	12 (3.8%)			0.468
Chronic kidney disease	2 (1.0%)	4 (1.3%)			1.000
History of stroke	3 (1.6%)	4 (1.3%)			1.000
Coronary artery disease	7 (3.6%)	12 (3.8%)			0.930
Previous cardiac surgery	10 (5.2%)	12 (3.8%)			0.449
Coronary malperfusion	14 (7.3%)	25 (7.9%)			0.799
Acute renal failure	3 (1.6%)	4 (1.3%)			1.000
Bowel ischemia	2 (1.0%)	4 (1.3%)			1.000
Lower limb ischemia	7 (3.6%)	14 (4.4%)			0.667
Cardiac tamponade	13 (6.8%)	18 (5.7%)			0.624
Maximum aortic diameter^b^ (mm)	38.5 ± 3.5	39.0 ± 2.9	0.200/0.200	0.002	0.143
**Affected coronary artery**					0.693
No	119 (62.0%)	207 (65.5%)			
Neri type A	55 (28.6%)	80 (25.3%)			
Neri type B	18 (9.4%)	29 (9.2%)			
LVEF (%)	64.0 ± 3.1	63.5 ± 4.2	0.088/0.062	0.001	0.103
**Aortic valve regurgitation**					0.368
None	75 (39.1%)	107 (33.9%)			
Mild	80 (41.7%)	134 (42.4%)			
Moderate	37 (19.3%)	75 (23.7%)			

a The grades of hypertension were classified by 2013 Practice guidelines for the management of arterial hypertension of the European Society of Hypertension (ESH) and the European Society of Cardiology (ESC) (15).

b In the aortic sinus.

RG, repair group; BG, Bentall group; LVEF, left ventricular ejection fraction.

### Surgical data

Operative details and concomitant procedures of the groups are provided in Table 2. The cardiopulmonary bypass time and cross-clamp time of the RG were both significantly less than those of the BG.

**Table 2:** Operative details of and concomitant procedures performed for the patients.

	**RG** **(*n* = 192)**	**BG** **(*n* = 316)**	**Kolmogorov–Smirnov test (RG/BG)**	**Levene's test**	***P*-value**
Operative time (min)	319.5 ± 40.5	362.3 ± 42.5	0.076/0.073	0.729	0.000
CPB time (min)	200.8 ± 33.3	255.7± 32.5	0.200/0.055	0.865	0.000
Cross-clamp time (min)	100.9 ± 19.5	152.5 ± 20.7	0.200/0.200	0.664	0.000
DHCA time (min)	17.4 ± 3.8	17.9 ± 3.5	0.058/0.200	0.173	0.106
**Concomitant procedure**					
Coronary artery bypass graft	25 (13.0%)	45 (14.2%)			0.699
Mitral valve repair	10 (5.2%)	19 (6.0%)			0.705
Tricuspid valve repair	4 (2.1%)	7 (2.2%)			1.000

RG, repair group; BG, Bentall group; CPB, cardiopulmonary bypass; DHCA, deep hypothermic circulatory arrest.

### Early outcomes

In the RG, eight patients died before hospital discharge, resulting in an early mortality rate of 4.2% (8/192). The causes of death included heart failure (1 patient), cerebral hemorrhage (2 patients), gastrointestinal hemorrhage (1 patient), sepsis (2 patients), and multiple organ dysfunction syndrome (2 patients). Repeat exploration was performed for bleeding, heart failure, cerebrovascular event, gastrointestinal hemorrhage, pneumonia, postoperative renal failure, and multiple organ dysfunction syndrome for 4 (2.1%), 2 (1.0%), 10 (5.2%), 5 (2.6%), 7 (3.6%), 6 (3.2%; five patients with renal failure before surgery had been excluded), and 2 (1.0%) patients, respectively.

In the BG, 16 patients died before hospital discharge, resulting in an early mortality rate of 5.1% (16/316). The causes of death included heart failure (one patient), cerebral hemorrhage (four patients), gastrointestinal hemorrhage (four patients), sepsis (two patients), and multiple organ dysfunction syndrome (five patients). Repeat exploration was performed for bleeding, heart failure, cerebrovascular event, gastrointestinal hemorrhage, pneumonia, postoperative renal failure, and multiple organ dysfunction syndrome for 8 (2.5%), 3 (1.0%), 18 (5.2%), 13 (4.1%), 10 (3.2%), 24 (7.8%; eight patients with renal failure before surgery had been excluded), and 5 (1.6%) patients, respectively. The incidence of postoperative renal failure in the BG was significantly higher than that of the RG, and no significant differences between the two groups regarding other early outcomes were identified (Table 3).

**Table 3:** Hospital mortality and morbidity.

	**RG** **(*n* = 192)**	**BG** **(*n* = 316)**	***P*-value**
Hospital mortality	8 (4.2%)	16 (5.1%)	0.644
Repeat exploration for bleeding	4 (2.1%)	8 (2.5%)	0.983
Heart failure	2 (1.0%)	3 (1.0%)	1.000
Cerebrovascular event	10 (5.2%)	18 (5.7%)	0.815
Gastrointestinal hemorrhage	5 (2.6%)	13 (4.1%)	0.372
Pneumonia	7 (3.6%)	10 (3.2%)	0.770
Postoperative renal failure^a^	6 (3.1%)	24 (7.6%)	0.038
MODS	2 (1.0%)	5 (1.6%)	0.909

a Patients with renal failure before surgery were excluded.

RG, repair group; BG, Bentall group; MODS, multiple organ dysfunction syndrome.

### Late outcomes

**Figure 3:**
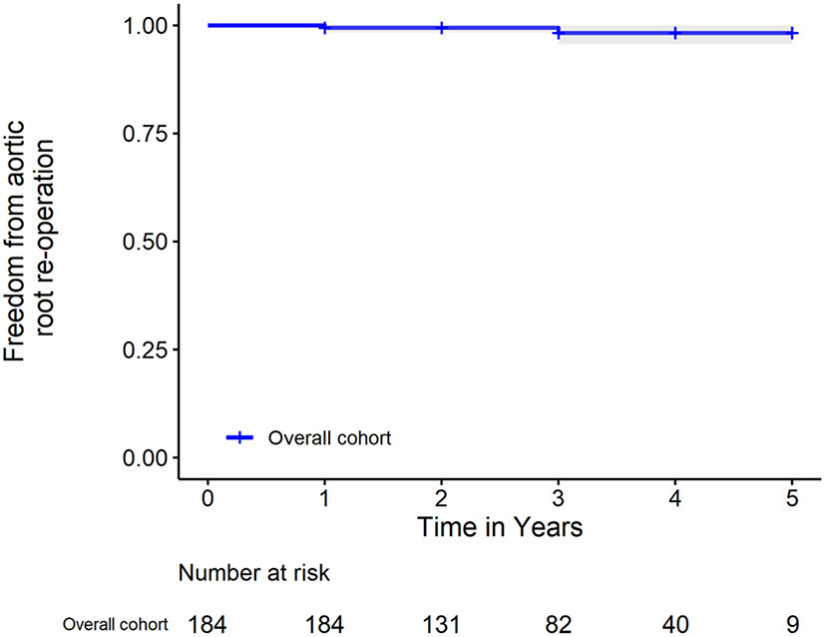
Kaplan-Meier analysis of freedom from aortic root reoperation. The shadow area represents the 95% confidence interval.

**Figure 4:**
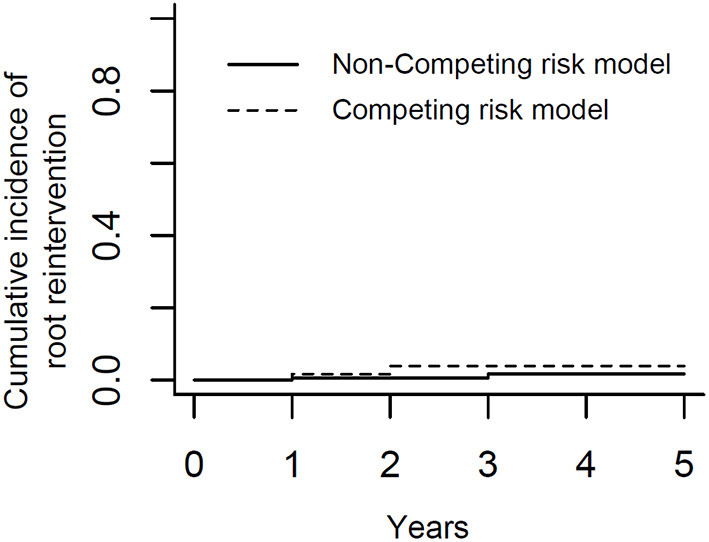
Competing risk analysis of freedom from aortic root reoperation.

After discharge, all surviving patients underwent follow-up at 1, 3, 6, 12 months, and yearly thereafter. The median follow-up time was 2 years (range, 1–5 years). In the RG, six patients died during follow-up. Causes of late deaths were descending aortic rupture (*n* = 1), stroke (*n* = 2), malignant tumor (*n* = 1), and sepsis (*n* = 2). The mortality rate related to ATAAD was 6.8% (13/191) because the data of one patient were censored. Two patients underwent aortic root reoperation because of an aortic root pseudoaneurysm. Based on result of K-M analysis (Figure 3), the rate of freedom from aortic root reoperation at 5 years was 98.2%. To obviate interference of death, we also performed competing risk analysis and showed it in Figure 4. The result revealed that there was no significant difference between competing risk analysis and K-M analysis. No patients had severe aortic valve regurgitation. The aortic regurgitation grades at 6 months later were significantly lower than those before surgery, and aortic valve regurgitation did not worsen during later follow-up (Table 4). The maximum aortic diameter of the aortic sinus at 6 months later was also significantly smaller than that before surgery, and it did not become larger during later follow-up (Table 4). In the BG, 14 patients died during follow-up. Causes of late deaths were descending aortic rupture (*n* = 1), stroke (*n* = 3), malignant tumor (*n* = 2), visceral hemorrhage (*n* = 5), and sepsis (*n* = 3). The mortality rate related to ATAAD was 8.9% (28/314) because the data of two patients were censored. Only one patient underwent aortic root reoperation because of an aortic root pseudoaneurysm. There were no significant differences between the two groups regarding ATAAD-related mortality (*P* = 0.400) and reoperation (*P* = 0.668).

**Table 4:** Aortic valve regurgitation and diameter of the aortic root of patients in the repair group.

**6 months later vs. before surgery**				
	**Before surgery**	**6 months later**	**Kolmogorov–Smirnov test**	***P*** **-value**
**Aortic valve regurgitation**				0.000
None	74 (40.2%)	80 (43.5%)		
Mild	76 (41.3%)	96 (52.2%)		
Moderate	34 (18.5%)	8 (4.3%)		
Maximum aortic diameter^a^ (mm)	38.5 ± 3.5	33.2 ± 3.2	0.002	0.000
**1 year later vs. 6 months later**				
	**6 months later**	**1 year later**	**Kolmogorov–Smirnov test**	***P*** **-value**
**Aortic valve regurgitation**				0.603
None	79 (43.2%)	70 (38.3%)		
Mild	96 (52.5%)	103 (56.3%)		
Moderate	8 (4.4%)	10 (5.5%)		
Maximum aortic diameter^a^ (mm)	33.2 ± 3.1	33.7 ± 4.5	0.200	0.062
**3 years later vs. 6 months later**				
	**6 months later**	**3 years later**	**Kolmogorov–Smirnov test**	***P*** **-value**
**Aortic valve regurgitation**				0.479
None	29 (34.9%)	23 (27.7%)		
Mild	49 (59.0%)	52 (62.7%)		
Moderate	5 (6.0%)	8 (9.6%)		
Maximum aortic diameter^a^ (mm)	33.5 ± 2.9	34.2 ± 5.0	0.200	0.122

a In the aortic sinus.

## Discussion

For most patients with ATAAD, the primary intimal tear is situated on the ascending aorta, with the dissection flap extending into the non-coronary sinus rather than the right and left coronary sinuses. The reason for aortic valve regurgitation in most patients with ATAAD is the unhinging of one or more of the aortic valve commissures caused by the disruption of the aortic wall; there is no organic disease in the aortic valve leaflets or annulus (16). Based on aforementioned pathophysiological mechanisms, we tried to preform valve-sparing aortic root reconstruction with a bovine pericardium patch for selected patients with ATAAD to reduce the complexity of the proximal procedure. Gelatin resorcin formalin glue, fibrinous glue, and Teflon felt strips were commonly used to repair the aortic root; however, the perioperative mortality rates of patients with ATAAD undergoing replacement of ascending aorta with aortic root preservation differ widely (6.6–34.0%) (8–10, 12). The early mortality rate during our study was 4.2%, which is within the lower end of the published range. Recently, more researchers have performed valve-sparing aortic root reimplantation (David procedure) for patients with ATAAD, and some of them have achieved low in-hospital mortality rates and low long-term reoperation rates. However, the David procedure is so difficult and complex that it is difficult popularize it at most hospitals. We think our novel technique is simple and effective and can be used to save the lives of selected patients with ATAAD at most cardiac surgery centers.

Successful reconstruction of the aortic sinus is dependent on the complete elimination of aortic sinus dissection, the prevention of aortic root bleeding, and the avoidance of aortic valve regurgitation caused by aortic sinus dilation and dissection or pseudoaneurysm, which are the major reasons for reoperation (17–21). Many surgical techniques have been reported, with the most frequent being proximal aortic stump reconstruction with resuspension of aortic valve (22). However, the incidence of reoperation was still high because of the fragile aortic tissue in patients with ATAAD. The rate of freedom from aortic root reoperation was only 81 to 96% at 5 years (17–21). It is necessary to continue exploring new technique to obtain better results. Rylski et al. (5) reported placing the Teflon between the dissected layers to stabilize the aortic root and the rate of freedom from aortic root reoperation was 92% at 10 years. However, it was really difficult to fashion the Teflon felt to suitably reconstruct the whole dissected aortic sinus. Tang et al. (9) recently reported placing the Teflon externally and internally around the dissected aortic wall to stabilize the aortic root; this procedure was successfully performed for most patients with ATAAD. The rate of freedom from aortic root reoperation was 100% at 5 years. However, the Teflon felt is much stiffer than bovine pericardium; therefore, it is more difficult to maneuver the Teflon felt to reconstruct the aortic sinus. Moreover, the stiff Teflon felt may result in more oozing of blood from needle holes and deformation of the aortic root in the future. Aortic root reconstruction with a bovine pericardium patch is simple and effective for preventing tears and bleeding from needle holes in the dissected tissue. During our research, the rate of freedom from aortic root reoperation at 5 years was 98.2%. Only two patients needed aortic root reoperation, which was performed in 2016 and 2017, respectively, when our novel surgical technique was still new. Furthermore, the aortic regurgitation grades at 6 months later were significantly lower than those before surgery, and aortic valve regurgitation did not worsen during later follow-up. The maximum aortic diameter of the aortic sinus at 6 months later was also significantly smaller than that before surgery, and it did not become larger during later follow-up. These results indicated that our novel aortic root repair technique was efficient for restoring the geometry of the aortic sinus and valve.

The Bentall procedure is the standard procedure for patients with aortic root dissection (6, 11). However, it is also relatively complex, and all patients should receive oral warfarin anticoagulation after surgery. During our research, clinical characteristics, operative data, mortality rates, and morbidity rates were compared between the RG and the BG. Because of our inclusion criteria, there were no significant differences in preoperative clinical characteristics, including maximum aortic diameter of the aortic sinus and aortic valve regurgitation, between these two groups. Because our novel surgical technique was simpler than the Bentall procedure, the operative time, cardiopulmonary bypass time and cross-clamp time of the RG were significantly shorter than those of the BG, which resulted in a higher incidence of postoperative renal failure in the BG. Oral warfarin anticoagulation resulted in visceral hemorrhage, including cerebral hemorrhage and gastrointestinal hemorrhage, and, subsequently, death for five patients in the BG. Although there were no significant differences in early and late mortality rates between these two groups, we thought that patients who underwent our novel technique would achieve better results if we could enroll more patients.

Our study had several limitations. First, the including criteria included the judgment and preference of the attending surgeon which made the research less convincing. Second, this was a retrospective study involving a single institution. The small number of patients and short follow-up time limited the statistical power of the research. Third, we did not perform learning curve analysis of our novel surgical technique and the statistical methods in this study seemed to be simple. More refined studies are necessary to determine the best surgical technique for aortic root reconstruction in patients with ATAAD.

## Conclusions

Valve-sparing aortic root reconstruction with a bovine pericardium patch can be successfully performed for selected patients with ATAAD and is associated with low in-hospital and late mortality rates and low aortic root reoperation rates during early and midterm follow-up.

## Data availability statement

The raw data supporting the conclusions of this article will be made available by the authors, without undue reservation.

## Ethics statement

The studies involving human participants were reviewed and approved by Research Ethnics Committee of Guangdong Provincial People's Hospital, Guangdong Academy of Medical Sciences. The patients/participants provided their written informed consent to participate in this study.

## Author contributions

JY, XL, and MW wrote the main manuscript text. JW and ZC prepared Tables 1–4. TS prepared Figures 1, 2. CY and RF were the managers of the whole study. All authors reviewed the manuscript. All authors contributed to the article and approved the submitted version.

## Funding

This research was supported by the National Key Research and Development Program of China (2017YFC1308003), the Science and Technology Program of Guangzhou (202102020160), and the Medical Scientific Research Foundation of Guangdong Province (A2022493). All the authors have nothing to disclose.

## Conflict of interest

The authors declare that the research was conducted in the absence of any commercial or financial relationships that could be construed as a potential conflict of interest. The handling editor ES declared a past co-authorship with the author RF.

## Publisher's note

All claims expressed in this article are solely those of the authors and do not necessarily represent those of their affiliated organizations, or those of the publisher, the editors and the reviewers. Any product that may be evaluated in this article, or claim that may be made by its manufacturer, is not guaranteed or endorsed by the publisher.
